# Structurally Colored Photonic Janus Films for Switchable Radiative Cooling and Solar Heating

**DOI:** 10.3390/nano16100603

**Published:** 2026-05-14

**Authors:** Wei Wei, Hengrui Gu, Xingchen Zhao, Yulong Lu, Kerui Li, Hongzhi Wang, Jianjun Zhang

**Affiliations:** 1 State Key Laboratory of Advanced Fiber Materials, College of Materials Science and Engineering, Donghua University, Shanghai 201620, China; better0122@163.com (W.W.); laiwuguhengrui@163.com (H.G.); zxc391728515@163.com (X.Z.); lyl100123@163.com (Y.L.); likr@dhu.edu.cn (K.L.); 2 Key Laboratory of High Performance Fibers & Products, Ministry of Education, Donghua University, Shangha 201620, China; 3 School of Material Science and Engineering, Shanghai Dianji University, Shanghai 201306, China; 4 School of Aeronautics, Shanghai Dianji University, Shanghai 201306, China

**Keywords:** photonic Janus films, structural color, radiative cooling, solar heating, switchable thermal regulation

## Abstract

Personal thermal management (PTM) requires materials that can adapt to dynamically changing environments, yet most existing systems are limited to single-mode cooling or heating and lack tunable optical appearance. Here, we report a structurally colored photonic Janus film that enables switchable radiative cooling and solar heating within a single material platform. The cooling side is based on cellulose nanocrystal (CNC) photonic structures, providing high mid-infrared emissivity (up to 0.91) while enabling tunable structural color through pitch modulation, thereby achieving radiative cooling without sacrificing visual appearance. The heating side consists of a carbon nanotube (CNT) layer with high solar absorptivity (~92%), enabling efficient photothermal conversion, further enhanced by low-voltage Joule heating. By simply flipping the film, reversible switching between cooling and heating modes is achieved, allowing adaptation to varying thermal conditions. In addition, the system is compatible with continuous fabrication, enabling scalable production. This work demonstrates a dual-mode photonic thermal management system that integrates optical tunability with switchable thermal regulation, providing a viable pathway toward wearable microclimate control.

## 1. Introduction

Thermal comfort is essential for maintaining human physiological function, and exposure to inappropriate thermal environments can lead to discomfort and even health risks [1,2]. In this context, personal thermal management (PTM), which directly regulates heat exchange between the human body and the surrounding environment, has emerged as an energy-efficient alternative to conventional space-conditioning systems [3,4,5,6]. Among various strategies, passive radiative cooling (PRC) and passive radiative heating (PRH) have attracted considerable attention due to their ability to modulate thermal radiation without external energy input [7,8,9,10,11,12,13].

PRC operates by combining high solar reflectance with strong mid-infrared emissivity within the atmospheric window (8–13 μm), enabling net radiative heat dissipation. Representative systems, including nanoporous polymer textiles and cellulose-based emitters, have demonstrated promising cooling performance for wearable applications [4,6]. In contrast, PRH relies on photothermal materials that efficiently absorb solar radiation while suppressing infrared emission to minimize heat loss [11,12,13]. Despite these advances, most existing radiative thermal management materials are limited to single-mode operation, with fixed optical and thermal properties once fabricated [11,14,15,16]. As a result, they cannot accommodate dynamically varying environmental conditions, where alternating cooling and heating demands are often required. To overcome this limitation, dual-functional systems integrating radiative cooling and heating have recently been explored. In particular, Janus-structured materials, which combine asymmetric optical properties on two opposing surfaces, enable mode switching through structural reorientation [17,18,19]. However, current Janus systems still face several challenges. Their fabrication often relies on complex multilayer processes such as vacuum deposition or sputtering, limiting scalability and increasing cost [6,12]. In addition, most reported systems are dominated by inorganic materials (e.g., SiO_2_ and TiO_2_), restricting material diversity and flexibility [4,20]. More importantly, the aesthetic limitation of radiative cooling materials remains largely unresolved, as achieving high solar reflectance typically requires white or non-tunable surfaces, which constrains their practical use in wearable and consumer-oriented scenarios [21,22,23].

Addressing these challenges requires a material system that simultaneously enables scalable fabrication, adaptive thermal regulation, and tunable optical appearance. Cellulose nanocrystals (CNCs) provide a promising platform in this regard. Owing to their abundant polar functional groups, CNCs exhibit intrinsically high mid-infrared emissivity, making them suitable for radiative cooling [23,24]. More importantly, CNCs can self-assemble into chiral nematic photonic structures that produce structural color via selective reflection, a phenomenon also observed in natural systems such as chameleons, allowing optical tunability without significantly compromising thermal performance [25,26]. This unique combination offers a pathway to integrate radiative cooling with controllable visual appearance. For the heating component, carbon nanotube (CNT) films are attractive due to their broadband solar absorption and low infrared emissivity, enabling efficient photothermal conversion while reducing radiative heat loss [27,28,29]. Their high electrical conductivity further allows the incorporation of low-voltage (2–4 V) Joule heating compatible with portable wearable electronic systems, extending the functionality to active thermal regulation [30,31].

**Figure 1:**
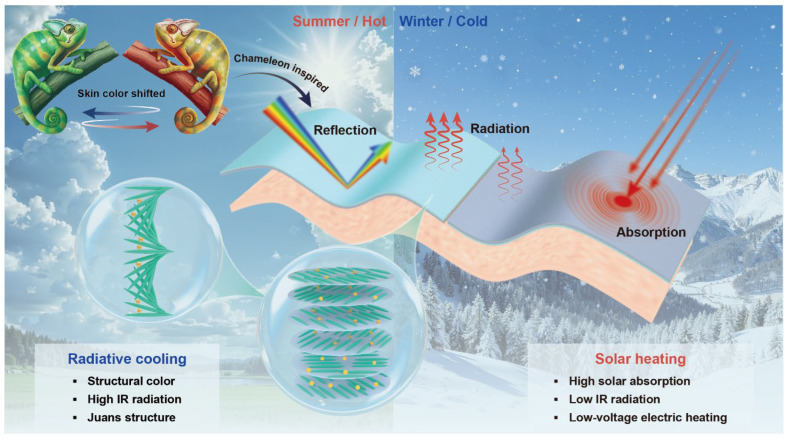
Schematic diagram of a photonic Janus film with switchable radiation cooling and solar heating.

Here, we present a structurally colored photonic Janus film (termed C/H Janus film) for switchable radiative cooling and solar heating (Figure 1 and Figure S1). The film integrates a CNC-based photonic cooling layer with tunable structural color and high infrared emissivity, and a CNT-based heating layer featuring high solar absorptivity and electrical conductivity. This combination enables dual-mode thermal regulation within a single material system, where cooling and heating can be switched simply by flipping the film. In addition to passive solar heating, the conductive CNT layer also supports active Joule heating under low operating voltages, thereby extending the thermal regulation capability for wearable applications. A continuous fabrication strategy compatible with roll-to-roll processing is established, enabling scalable production of the Janus films. Beyond dual-mode functionality, the CNC layer provides a structurally tunable photonic platform, allowing the optical response to be modulated through controlled self-assembly. This feature, combined with the asymmetric spectral design of the Janus structure, enables effective thermal regulation through balanced control of solar absorption and infrared emission. As a result, the C/H Janus film achieves integrated optical tunability and switchable thermal management without compromising performance, offering a viable pathway toward wearable microclimate regulation under dynamic environmental conditions.

## 2. Materials and Methods

Materials: The cellulose nanocrystal (CNC) suspension (3 wt.%) was purchased from Nanjing Ruiniu Nenghuan New Material Technology Co., Ltd. (Nanjing, China). Glycerol was obtained from Aladdin Reagent Co., Ltd. (Shanghai, China). The multi-walled carbon nanotube (MWCNT) film (~10 μm thick) was purchased from JIACAI Technology Co., Ltd. (Chengdu, China). The film exhibits an interconnected porous microstructure (Figure S2) and high electrical conductivity of approximately 812 S cm^−1^ at room temperature (300 K, Figure S3), enabling efficient electrical transport and facilitating precursor infiltration during coating.

Preparation of Janus Films: Glycerol was added to the CNC suspension at a predetermined mass ratio and ultrasonicated (40% power) for 5 min. The mixture was then magnetically stirred at room temperature for 12 h to obtain a homogeneous CNC/glycerol solution. The resulting solution was deposited onto a CNT film and dried under ambient conditions, yielding C/H Janus films with different structural colors.

Fabrication of Large-Scale Janus Films: For large-scale fabrication, a continuous slot-die coating process was employed. The CNT substrate was first transported at a controlled speed and subjected to corona discharge treatment to remove surface contaminants and improve wettability, thereby enhancing interfacial adhesion. The CNC-based coating solution was then delivered into the slot-die head using a peristaltic pump and uniformly coated onto the moving substrate at a preset thickness. The coated film subsequently passed through a semi-enclosed drying oven, where it was heated to accelerate solvent evaporation. After drying, the Janus film was continuously collected by a winding system.

Characterization and Measurements: SEM images were obtained using a scanning electron microscope (SU8010, Hitachi, Tokyo, Japan). Infrared reflectance and transmittance were measured using a Fourier-transform infrared spectrometer (Nicolet 6700, Thermo Fisher, Waltham, MA, USA), and the emissivity was calculated accordingly. Reflectance in the wavelength range of 0.25–2.5 μm was measured using a UV–Vis–NIR spectrophotometer (UHJ5700, Hitachi, Tokyo, Japan). Solar irradiance was measured under direct sunlight on a rooftop using a solar power meter (SOLAR-1, Jinzhou TINEL Environment Energy Instrument Co., Ltd., Jinzhou, China). Infrared thermal images were recorded using a thermal imager (A300, FLIR, Wilsonville, OR, USA). Temperature measurements were recorded using a thermocouple thermometer (TA612C, TASI, Suzhou, China, accuracy ±0.5 °C). The temperature data were continuously acquired at a sampling interval of 1 s over extended durations under outdoor conditions, resulting in temperature evolution profiles.

## 3. Results and Discussion

### 3.1. Design and Scalable Fabrication of Photonic Janus Films

**Figure 2:**
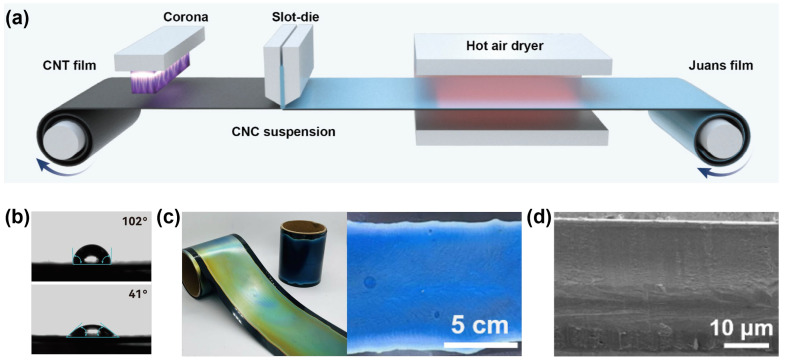
(**a**) Schematic diagram of the scalable continuous preparation process of C/H Janus film. (**b**) Demonstration of the hydrophilic property of CNT film after corona discharge treatment. (**c**) Physical display of large-area C/H Janus film. (**d**) SEM of the vertical section of C/H Janus film.

Figure 2a illustrates a continuous and scalable fabrication strategy for photonic Janus films via slot-die coating, a process inherently compatible with roll-to-roll manufacturing. This approach bridges the gap between laboratory-scale photonic materials and industrial production. It enables uniform, high-throughput deposition over large areas and overcomes the scalability limitations of conventional batch-based methods. A critical challenge in constructing the Janus architecture lies in the interfacial incompatibility between the hydrophobic CNT substrate and the hydrophilic CNC precursor solution. To address this issue, a corona discharge treatment was applied to modify the surface wettability of the CNT film prior to coating. This method is advantageous due to its open-air operation and compatibility with continuous processing. After treatment, the water contact angle of the CNT surface decreases markedly from 102° to 41.3°, indicating a transition from hydrophobic to hydrophilic behavior (Figure 2b). This substantial increase in surface energy promotes the spreading and adhesion of the CNC precursor, enabling the formation of a stable coating layer.

Benefiting from the low viscosity and excellent film-forming capability of the CNC precursor, a uniform coating can be continuously deposited onto the corona-treated CNT substrate and subsequently dried to form a well-defined Janus structure. This process enables the scalable production of large-area films with consistent structural integrity and surface quality (Figure 2c). SEM images (Figure 2d) show that the CNC layer is tightly integrated with the CNT substrate, forming a compact and well-bonded interface that effectively prevents delamination and ensures mechanical stability during use. The enhanced interfacial adhesion can be attributed not only to the improved surface wettability induced by corona treatment, but also to the porous structure of the CNT film, which enables partial infiltration of the CNC precursor and promotes mechanical interlocking at the interface (Figure S2). Tape-peeling tests further confirm the improved interfacial stability after corona treatment. As shown in Figure S4, the CNC coating on untreated substrates was almost completely removed after peeling, whereas the corona-treated samples maintained an intact coating morphology. In addition, the corona-treated films remained mechanically stable under bending deformation, demonstrating good flexibility and adhesion robustness. The photonic structural color of the CNC layer can be systematically tuned through process parameters such as coating thickness and drying conditions, linking fabrication directly to optical functionality.

### 3.2. Photonic Structural Color and Tunable Optical Properties

**Figure 3:**
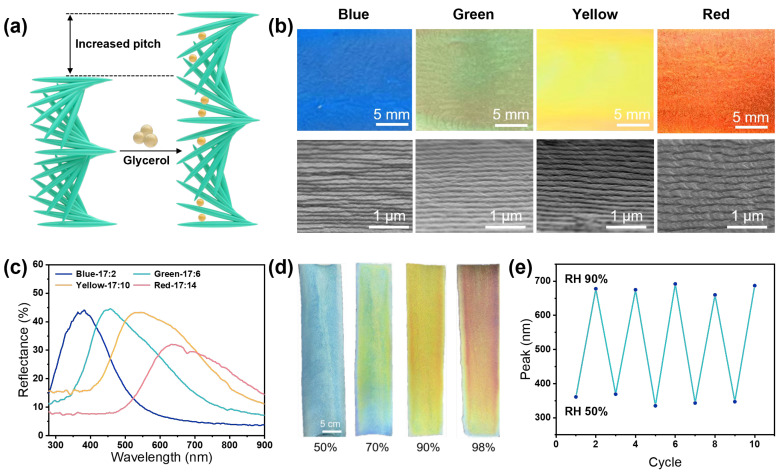
(**a**) Schematic diagram illustrating the mechanism by which the helical pitch of the CNCs films is increased through glycerol molecules. (**b**) Optical microscopic images of blue, green, yellow and red structural colors, as well as SEM images of the pitch variations of nanocellulose crystals. (**c**) Visible light reflection spectra of different ratios of CNCs films. As the proportion of glycerol added increases, the red shift phenomenon occurs in the CNCs films. (**d**) Color changes of CNCs films under different humidity levels. (**e**) In the cyclic test conducted at 50% and 90% humidity, the reflection peaks of the CNCs films remained relatively stable.

Highly concentrated cellulose nanocrystal (CNC) suspensions can self-assemble into photonic crystals with a chiral nematic (cholesteric) structure (Figure S5). This periodic helical arrangement generates a photonic bandgap in the visible range, enabling selective Bragg reflection of specific wavelengths and giving rise to structural color. According to the Bragg reflection equation [32], the peak reflection wavelength is determined by the helical pitch (*P*) and the average refractive index of the system.

(1) $$\lambda =n_{av}P\sin\theta$$

where *λ* is the peak reflection wavelength, $n_{av}$ is the average refractive index of the system, and P is the helical pitch of the chiral nematic structure. Therefore, the reflected wavelength increases with increasing helical pitch. By tuning these structural parameters during self-assembly, precise control over the resulting structural color can be achieved. This provides a structurally tunable platform for photonic color engineering. Based on this mechanism, glycerol was introduced as a structural modulation unit to regulate the photonic structure. Rich in hydroxyl groups, glycerol forms hydrogen bonds with CNC and becomes incorporated into the chiral liquid crystalline framework during self-assembly. This interaction increases the interlayer spacing, leading to an enlarged helical pitch and a corresponding redshift of the reflection peak (Figure 3a). As a result, a direct relationship between composition, structure, and optical response is established, enabling predictable and tunable structural color.

Specifically, CNC suspensions were mixed with glycerol at mass ratios of 17:2, 17:6, 17:10, and 17:14 (CNC suspension/glycerol), followed by coating and drying to obtain films exhibiting blue, green, yellow, and red structural colors, respectively. Considering the CNC concentration of 3 wt.%, the corresponding dry CNC/glycerol mass ratios in the final films were approximately 0.51:2, 0.51:6, 0.51:10, and 0.51:14, respectively, assuming complete removal of water during drying. Cross-sectional SEM images (Figure 3b) show that the periodicity of the chiral layered structure increases with glycerol content. Correspondingly, the reflection spectra exhibit a systematic redshift within the range of 360–630 nm (Figure 3c), consistent with the structural evolution. X-ray diffraction (XRD) results (Figure S6) confirm that the introduction of glycerol does not disrupt the intrinsic crystalline structure of cellulose, as all samples retain the characteristic diffraction peaks.

In addition to optical tunability, the system shows improved mechanical adaptability. The incorporation of glycerol provides a plasticizing effect, increasing the elongation at break from 0.6% to 2.7% (Figure S7), thereby enhancing flexibility and suitability for wearable applications. The photonic system also exhibits dynamic responsiveness to environmental humidity. Due to the hygroscopic nature of glycerol, the films undergo reversible swelling under varying humidity, leading to changes in the helical pitch and corresponding shifts in the reflection peak (Figure S8a). Macroscopically, this is observed as a gradual color transition from blue to red (Figure 3d). Consistent with this behavior, the humidity-induced variation primarily affects a narrowband reflection in the visible range, while its influence on thermal performance is expected to be limited, as radiative cooling is mainly governed by mid-infrared emissivity. The process remains stable over multiple humidity cycles, with no significant shift in the reflection peak at the same humidity level (Figure 3e), indicating reliable reversibility. Based on the tunable structure and environmental responsiveness, spatial control of structural color can be achieved through patterning. Deionized water and 10 wt.% glucose solutions were used as local modulation media to create controllable patterns on the film surface (Figure S8b). The structural changes induced by water are fully reversible upon evaporation, restoring the original color. In contrast, glucose molecules partially remain within the liquid crystalline structure, maintaining an enlarged local pitch and resulting in irreversible color fixation. This dual-mode regulation, combining reversible and irreversible responses, provides a strategy for stable structural color display and functional integration.

### 3.3. Spectral Properties and Radiative Cooling Performance

**Figure 4:**
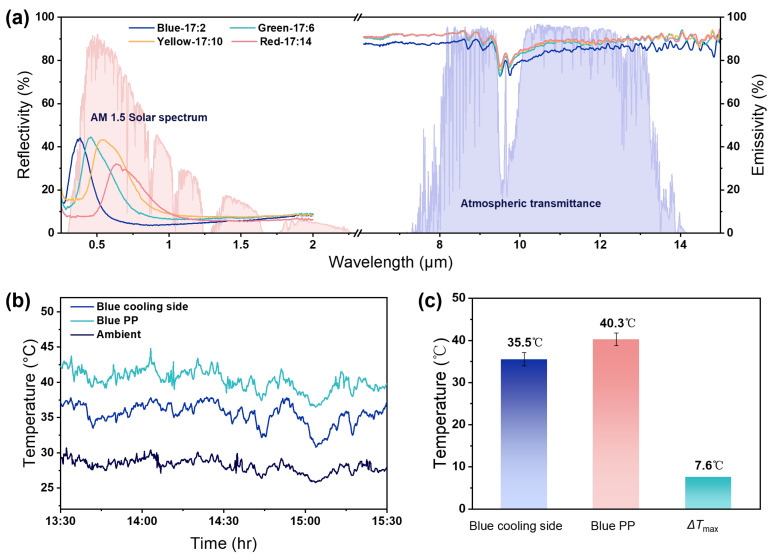
(**a**) Spectral performance of the cooling side of C/H Janus films with different structural colors. (**b**) Comparison of outdoor cooling performance between the blue C/H Janus film cooling side and ordinary blue PP fabric. (**c**) Comparison of the average temperature and maximum temperature difference of the samples during the test. The error bars represent the standard deviation of the average temperature measurements for the Janus film (cooling side) and the PP fabric, calculated from the data shown in (**b**).

The CNC film exhibits strong thermal radiation capability in the mid-infrared region, arising from the vibrational absorption of abundant polar functional groups (such as O–H, C–O, and C–O–C) in its molecular structure (Figure S9). These vibrational modes are well aligned with the mid-infrared range, particularly the atmospheric transparency window of 8–13 μm, enabling efficient outward thermal radiation. The characteristic absorption bands of the C–O bond (~1043 cm^−1^) and the C–O–C bond (~730 cm^−1^) contribute significantly to emission in this spectral range, resulting in an average emissivity of 0.86 for the pristine CNC film within the atmospheric window (Figure S10). Upon the introduction of glycerol, enhanced hydrogen bonding and a more compact structure lead to strengthened vibrational absorption in the mid-infrared region, increasing the emissivity to 0.91 (Figure 4a). In the solar spectral range, the optical response of the CNC coating is governed by its chiral nematic periodic structure, which induces selective reflection within specific wavelength ranges. This structural reflection reduces the effective absorption of incident solar radiation. As a result, the system combines high infrared emissivity with suppressed solar absorption. This spectral profile favors net heat loss: thermal energy is efficiently dissipated through radiation within the atmospheric window, while solar heat gain is limited, leading to a lower steady-state temperature under solar exposure.

To evaluate the radiative cooling performance, outdoor measurements were conducted under clear weather conditions in Shanghai (121°13′24 E, 31°02′4 N). The experimental setup was constructed based on previous reports (Figure S11). Measurements were performed on a rooftop between 13:30 and 15:30, using the blue C/H Janus film and a blue polypropylene (PP) sample as the control. During the test, the average solar irradiance was approximately 800 W m^−2^ (Figure S12). Under direct sunlight, the blue PP sample reached an average surface temperature of 40.3 °C (Figure 4b). In contrast, the C/H Janus film exhibited a lower steady-state temperature of 35.5 °C under identical conditions, approximately 4.8 °C lower than that of the same-colored PP, with a maximum temperature difference of up to 7.6 °C (Figure 4c). Based on the measured spectral properties of the cooling side (Figure 4a), the cooling power of the Janus film was theoretically calculated under different non-radiative heat transfer coefficients (*h*_non-rad_ = 0–8 W m^−2^ K^−1^) at an ambient temperature of 25 °C. Under the condition of $T_{s}-T_{amb}=0$, the cooling power reaches approximately 66.9 W m^−2^ (Figure S13). These results indicate that the material maintains spectral characteristics favorable for net radiative heat dissipation while enabling structural color tuning, demonstrating effective synergy between optical functionality and thermal management without significant performance trade-offs.

### 3.4. Solar Heating and Electrothermal Performance

**Figure 5:**
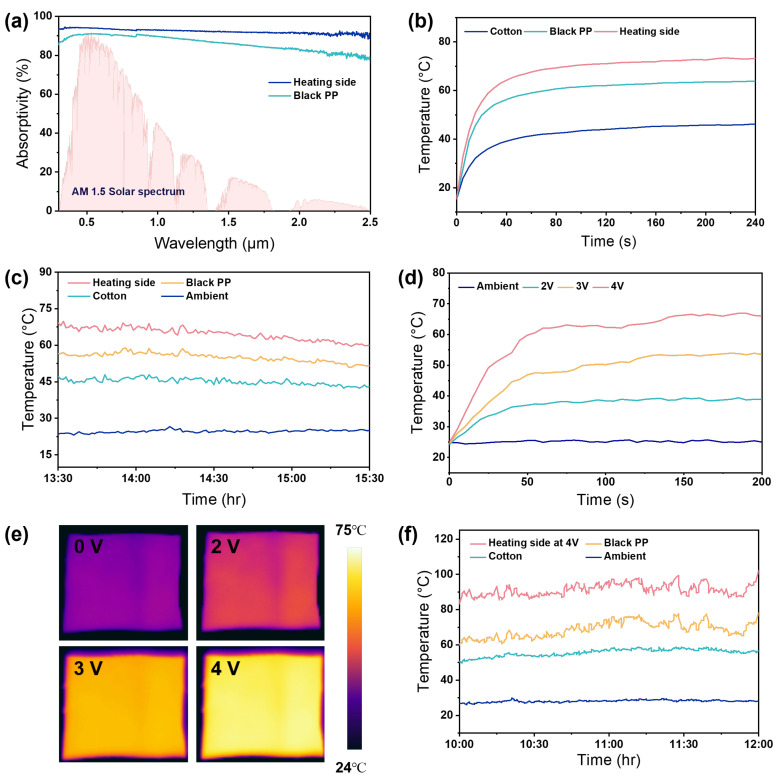
(**a**) Spectral performance of the heated side of C/H Janus film compared to conventional black PP fabric. (**b**) Temperature variations of different samples under the simulated standard solar light intensity of xenon lamps. (**c**) Outdoor passive heating performance test. (**d**) Temperature variations on the heated side of C/H Janus film under different applied low pressures. (**e**) Infrared thermal images of the heated side of C/H Janus film under different voltages. (**f**) Temperature variations of C/H Janus film heated side and conventional fabric under the combined effect of photothermal and electrical heating.

As shown in Figure 5a, the heating side of the C/H Janus film consists of a carbon nanotube (CNT) layer, which exhibits high solar absorptivity (~0.92), significantly higher than that of black polypropylene (PP) fabric. The CNT layer also shows moderate mid-infrared emissivity (~0.6), contributing to efficient photothermal conversion (Figure S14). This high absorptivity enables efficient photothermal conversion under solar irradiation. To evaluate the photothermal performance, a xenon lamp was used to simulate solar illumination under standard conditions. The C/H Janus film reaches a surface temperature of 76.2 °C (Figure 5b), which is 33.9 °C and 14.4 °C higher than those of cotton and PP fabrics, respectively. Under outdoor conditions (average solar irradiance ~800 W m^−2^, Figure S15), the heating side maintains an average temperature of 64.7 °C (Figure 5c), while cotton and black PP fabrics reach only 44.1 °C and 55.1 °C, respectively. Based on the measured spectral properties of the heating side (Figure 5a and Figure S15), the heating power was theoretically calculated. The heating power reaches approximately 521.8 W m^−2^ (Figure S16), indicating efficient photothermal conversion capability. These results indicate stable and efficient photothermal heating under realistic environmental conditions.

In addition to passive photothermal conversion, the CNT layer enables active Joule heating due to its high electrical conductivity (Figure S3). Under a low applied voltage of 2 V, the surface temperature increases to approximately 37 °C within ~50 s, corresponding to a temperature rise of ~12 °C relative to ambient conditions. With increasing voltage, the temperature further rises to approximately 65 °C at 4 V (Figure 5d), demonstrating effective heating under low driving voltage. The operating voltage range (2–4 V) is compatible with typical portable and wearable electronic power supplies, highlighting the practical applicability of the system for active thermal management. Infrared thermal imaging (Figure 5e) shows a uniform temperature distribution across the surface, which helps avoid localized overheating during practical use. Figure 5f presents the combined thermal response under simultaneous electrical and photothermal inputs. After reaching steady state, the heating side of the C/H Janus film attains a temperature of 92.1 °C, which is 22.2 °C and 36 °C higher than those of black PP and cotton fabrics, respectively. These results demonstrate that the combination of active and passive heating mechanisms enables substantial heat output, highlighting the effectiveness of the dual-mode heating strategy.

### 3.5. Outdoor Thermal Management Performance

**Figure 6:**
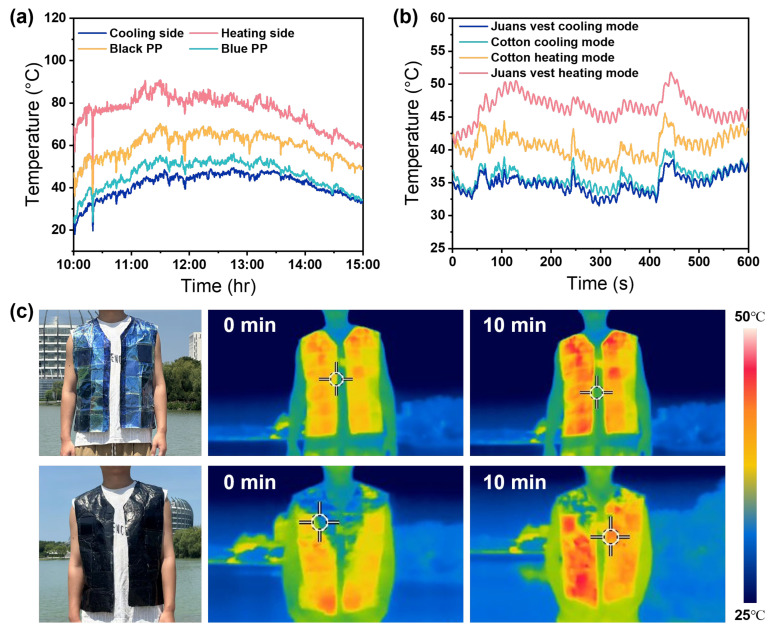
(**a**) The dual-mode outdoor thermal management performance of the C/H Janus film and its comparison with conventional fabrics. (**b**) The personal thermal management performance of the Janus vest made of the C/H Janus film. (**c**) Infrared thermal imaging of the Janus vest in an outdoor environment.

To evaluate the thermal management capability of the C/H Janus films under realistic wearing conditions, a constant-power heating platform (set at 35 °C) was used to simulate human skin. The samples were placed on the simulated surface, and their temperatures were recorded in real time using thermocouples. As shown in Figure 6a, the C/H Janus film exhibits pronounced temperature regulation under different operating modes, with steady-state temperature ranges of 17–40 °C for the cooling side and 50–90 °C for the heating side. Compared with polypropylene (PP) fabrics, the film shows an average temperature reduction of approximately 4.1 °C relative to blue PP in the cooling mode. In contrast, under the heating mode, the temperature is higher than that of blue and black PP fabrics by approximately 29.5 °C and 15.7 °C, respectively. Under high solar irradiation (peak intensity ~910 W m^−2^, Figure S17), the maximum temperature differences between the C/H Janus film and PP fabrics reach 10.2 °C in the cooling mode and 29.6 °C in the heating mode, further demonstrating its effective thermal regulation under dynamic environmental conditions.

To demonstrate practical applicability, a functional vest (Janus vest) was fabricated using the C/H Janus film and tested outdoors. After 10 min of heat exposure (Figure 6b), the vest maintains an average surface temperature of approximately 34.8 °C in the cooling mode, lower than that of cotton fabric (35.8 °C). In the heating mode, the temperature reaches 46.5 °C, about 5.9 °C higher than that of cotton. Infrared thermography (Figure 6c) further confirms effective regulation of body surface temperature under real wearing conditions. These results demonstrate that the C/H Janus film enables dual-mode thermal regulation through spectral control, allowing effective modulation of the human microclimate across varying environmental conditions. A quantitative comparison with recently reported Janus PTM systems is provided in Figure S18. It should be noted that the current demonstration focuses on thermal regulation performance, and the film has not yet been optimized for repeated washing or long-term mechanical durability.

## 4. Conclusions

In summary, we have developed a structurally colored photonic Janus film that integrates switchable radiative cooling and solar heating within a single material platform. By combining a CNC-based photonic cooling layer with a CNT-based heating layer, the system enables dual-mode thermal regulation through simple structural reorientation. The CNC layer provides high mid-infrared emissivity together with tunable structural color, establishing a direct link between photonic structure and optical functionality, while the CNT layer offers efficient photothermal conversion and low-voltage Joule heating capability. Importantly, a scalable fabrication strategy compatible with continuous processing is demonstrated, supporting large-area production of the Janus films. At the system level, the asymmetric spectral design enables balanced control of solar absorption and thermal radiation, resulting in effective heat dissipation or retention depending on the operating mode. This dual-mode behavior is further validated under realistic environmental and wearable conditions, where the film exhibits stable thermal regulation and practical applicability. However, the current system has not yet been optimized for washability and long-term durability, and further efforts on encapsulation strategies, textile integration, and standardized durability evaluation will be required for practical wearable applications. Overall, this work establishes a scalable and switchable material platform that integrates optical tunability with multifunctional thermal management, offering a viable pathway toward wearable microclimate regulation in dynamic environments.

## Data Availability

The data that support the findings of this study are available from the corresponding authors upon reasonable request.

## Supplementary Materials

The following supporting information can be downloaded at: https://www.mdpi.com/article/10.3390/nano16100603/s1. References [33,34,35,36,37,38,39,40,41,42] are cited in the Supplementary Materials.
