# Supplementary material for: Structurally Colored Photonic Janus Films for Switchable Radiative Cooling and Solar Heating
Source: Nanomaterials (Basel). 2026 May 14;16(10):603. doi: 10.3390/nano16100603 (PMC13209646; doi:10.3390/nano16100603)
Supplement: Supplementary file 1 [file nanomaterials-16-00603-s001.zip › nanomaterials-4306929-supplementary.pdf]

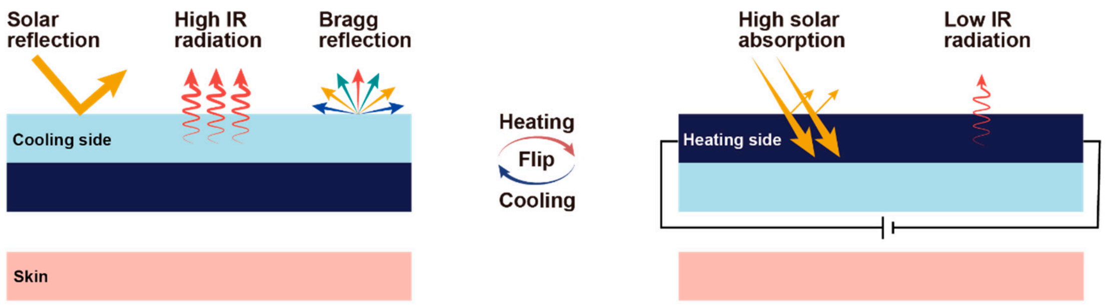

Figure S1. The operating mechanism principle of C/H Juans film achieving radiative cooling and solar heating (electric heating) through simple flipping.

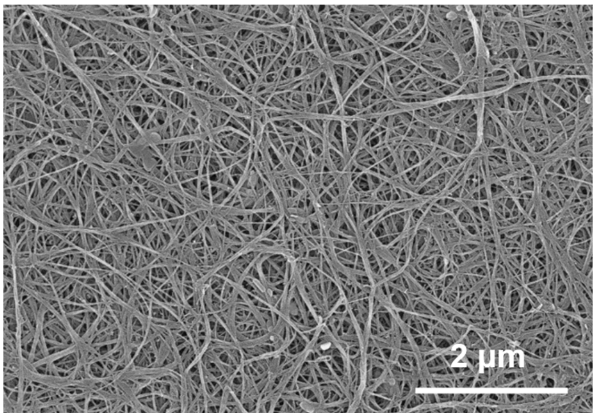

Figure S2. Morphological characterization of the CNT film.

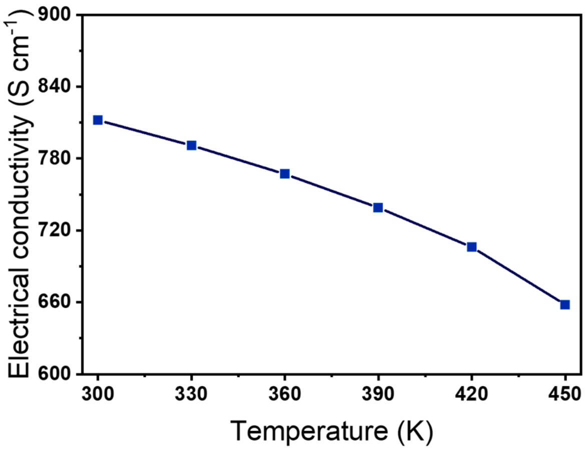

Figure S3. Temperature-dependent in-plane electrical conductivity.

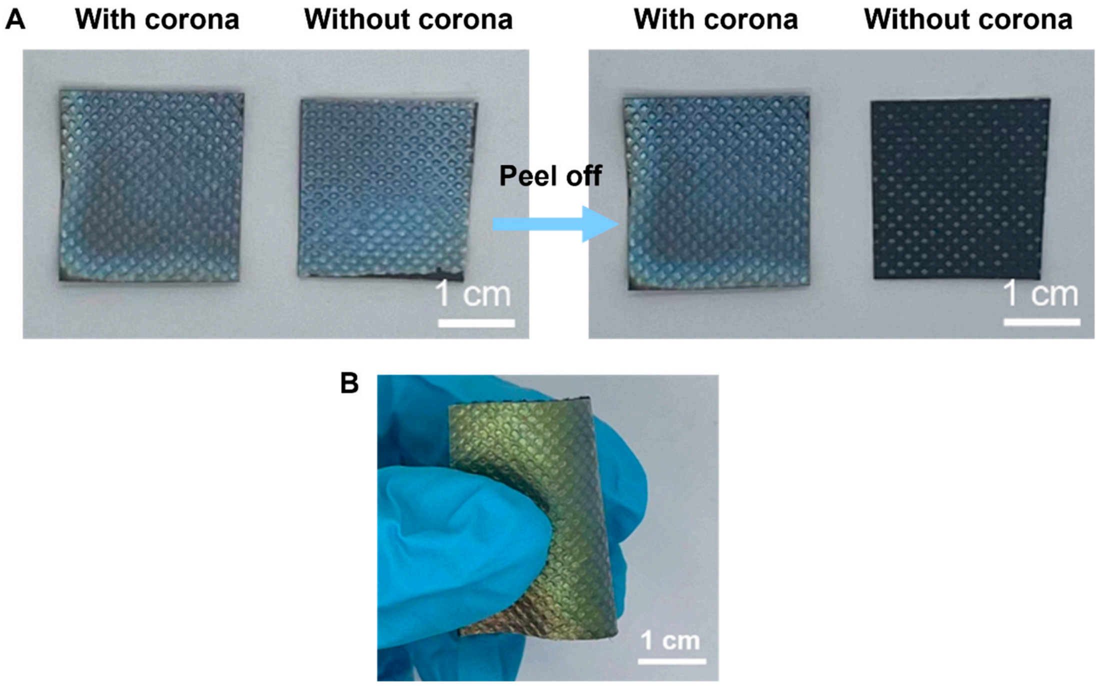

Figure S4. A) Comparison diagram of tape peeling for structured fabrics treated with corona discharge and those not treated with corona discharge. B) Photos of structural color fabric after bending.

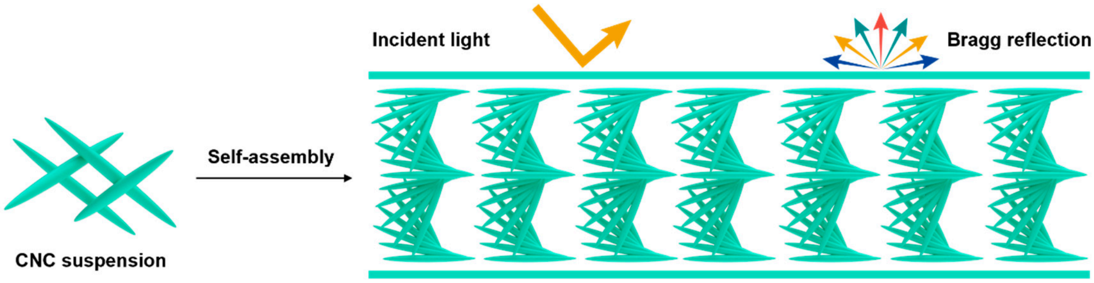

Figure S5. Schematic illustration of CNCs self-assembling into a chiral nematic (cholesteric) phase with a periodic helicoidal structure.

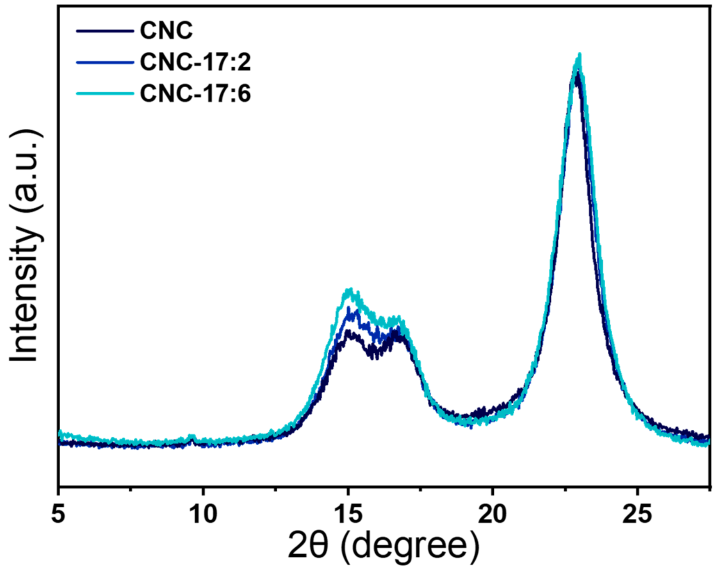

Figure S6. XRD profiles of CNCs and its composite films.

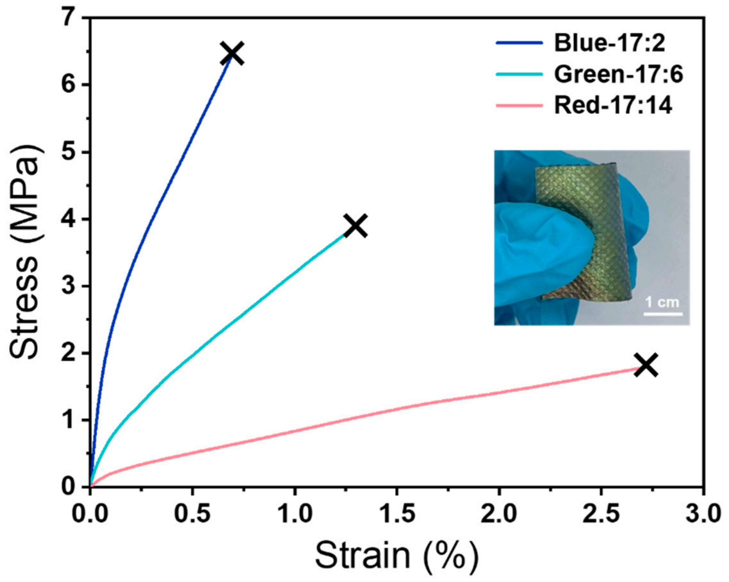

Figure S7. Tensile properties of CNCs films with different glycerin ratios added.

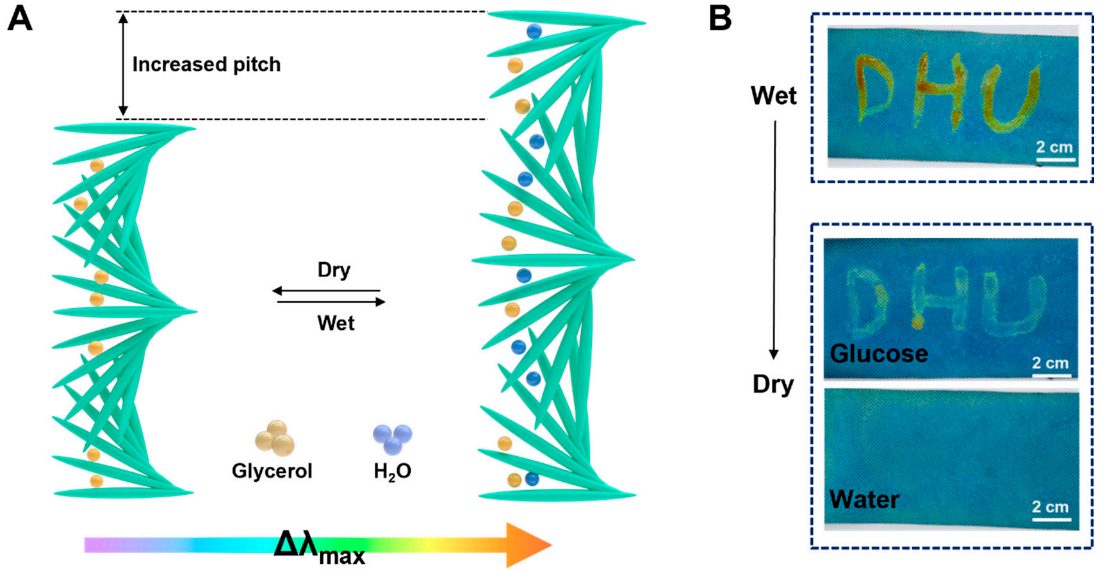

Figure S8. a) Schematic diagram illustrating the effect of swelling CNCs films (water and glucose) on pitch. b) Optical photographs obtained by patterning with water and glucose solutions respectively.

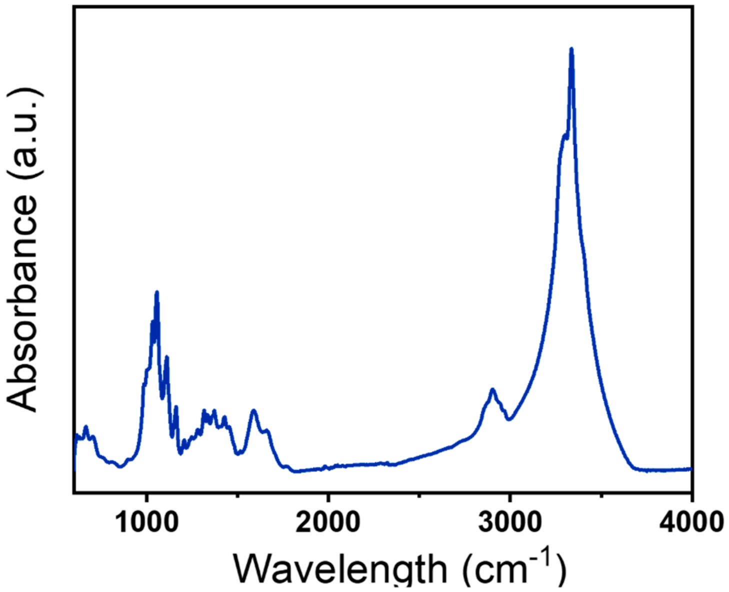

Figure S9. Infrared absorption spectrum of cellulose nanocrystals (CNCs).

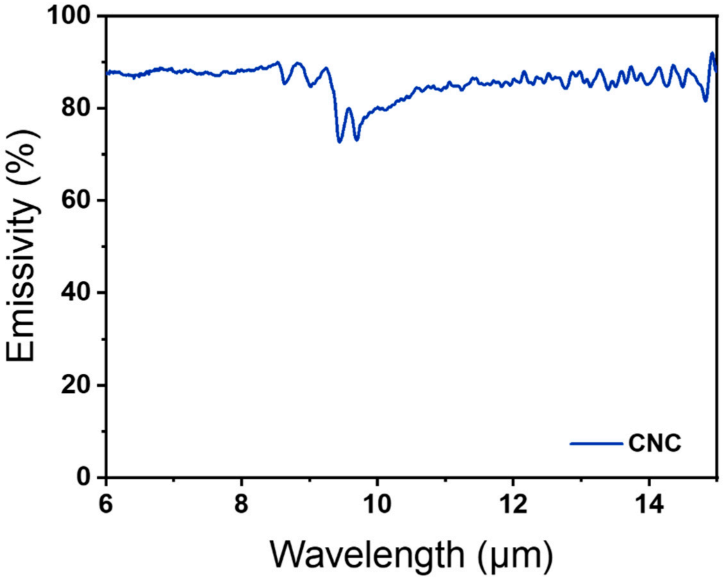

Figure S10. Mid-infrared emission spectrum of pure cellulose nanocrystals (CNCs).

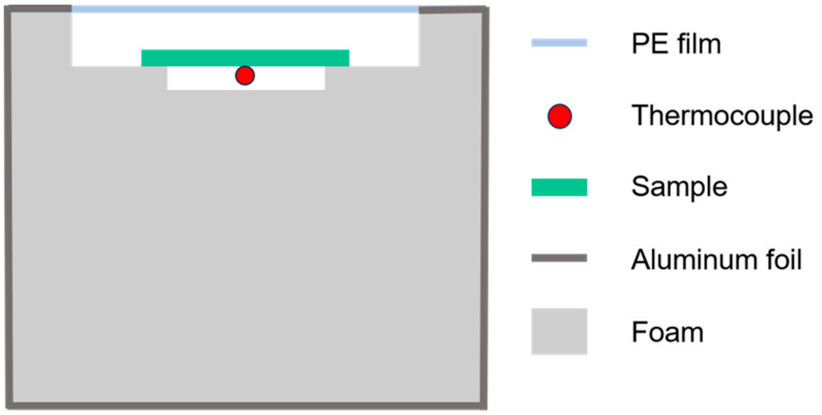

Figure S11. Experimental setup for outdoor radiation cooling performance test.

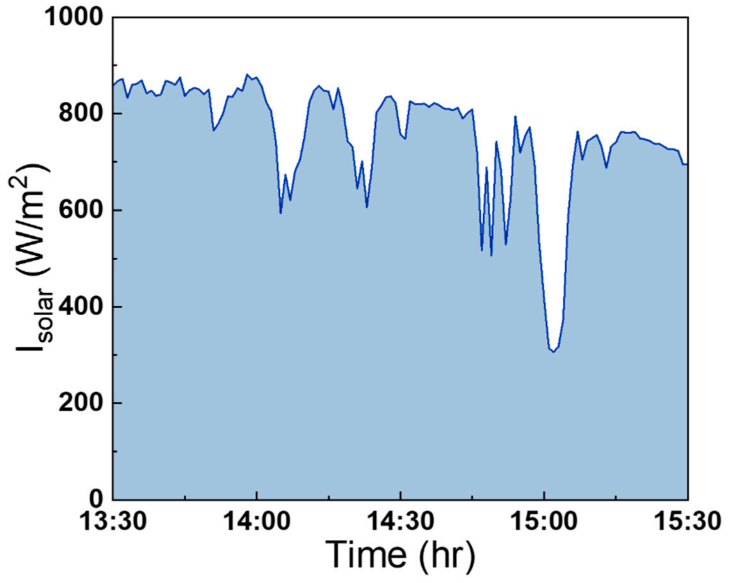

Figure S12. Solar irradiation intensity during the test period (corresponding to the cooling test conditions in Figure 4b).

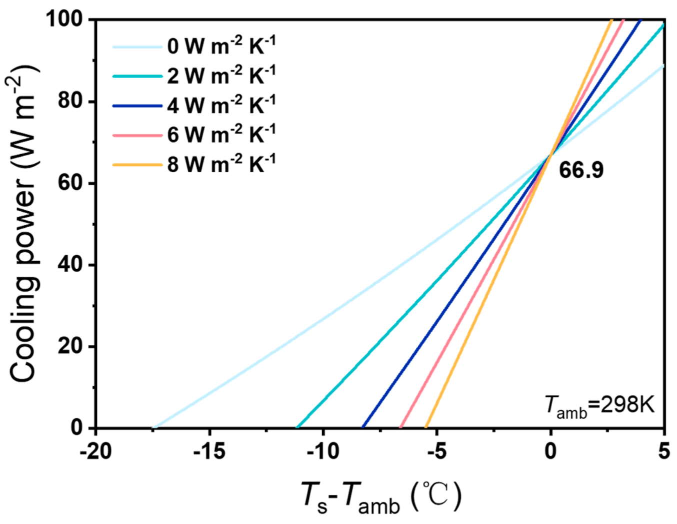

Figure S13. Calculated net cooling power of the cooling side of the Janus film.

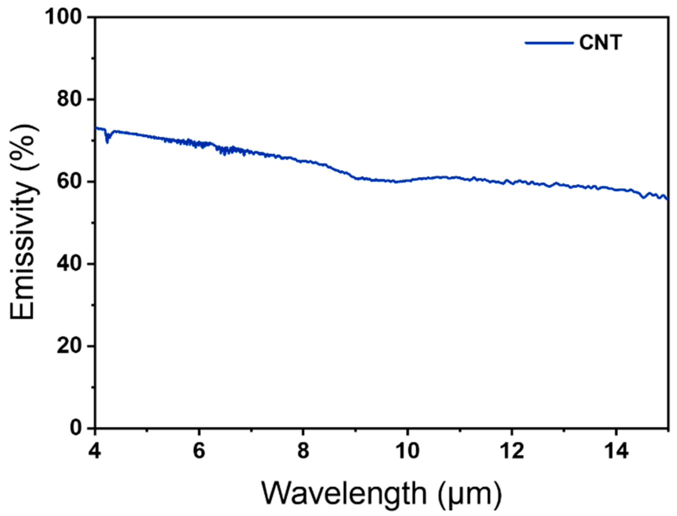

Figure S14. The mid-infrared (MIR) emissivity spectrum of the CNT heating layer.

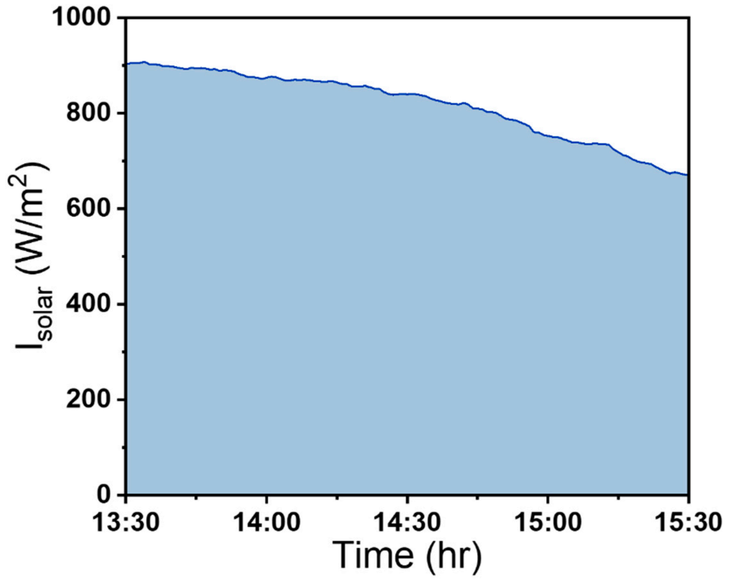

Figure S15. Solar irradiation intensity during the test period (corresponding to the cooling test conditions in Figure 5c).

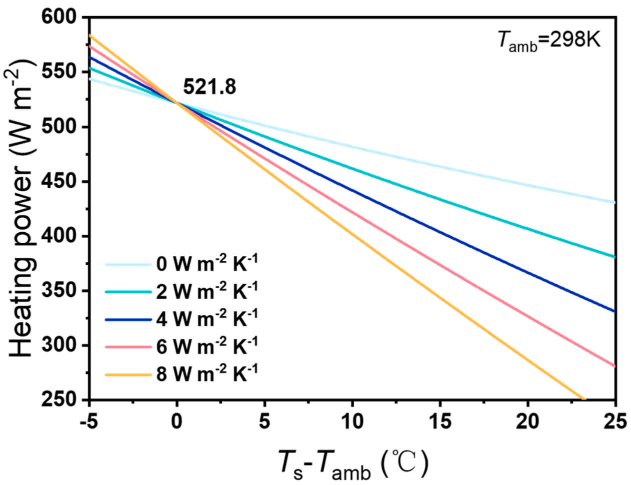

Figure S16. Calculated net heating power of the heating side of the Janus film.

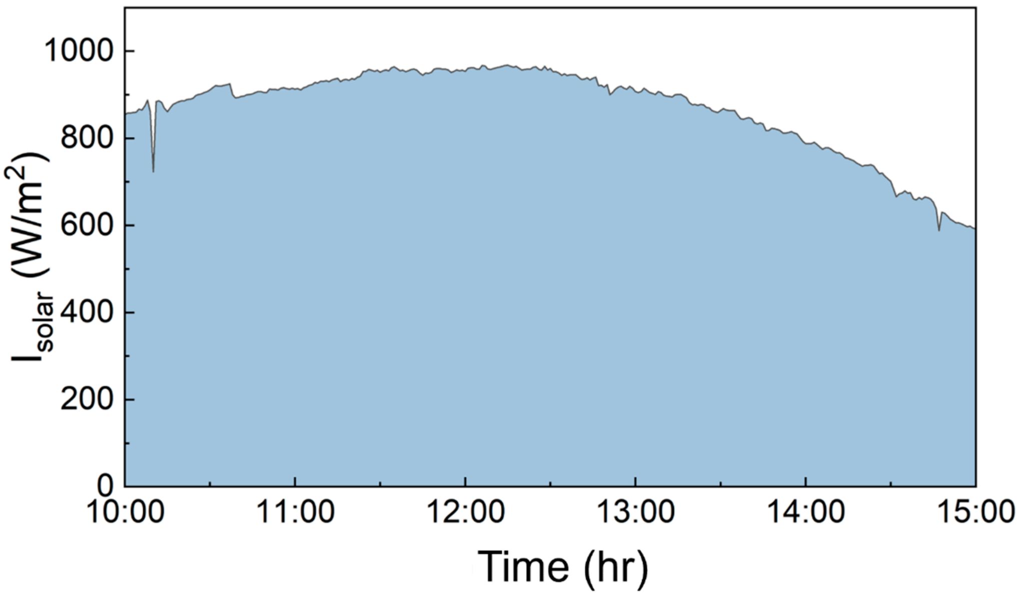

Figure S17. Solar irradiation intensity during the test period (corresponding to the cooling test conditions in Figure 6a).

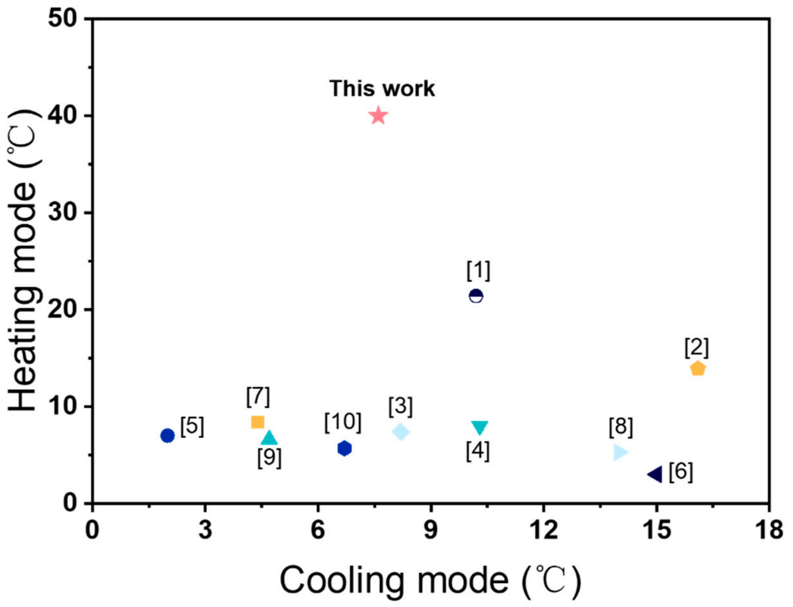

Figure S18. Quantitative comparison of the cooling-mode and heating-mode temperature differences between the present C/H Janus film and recently reported Janus PTM systems [1-10].

References:

[1] Tan J, Sun J, Han W, et al. Integrated cooling and heating regulation in colored photonic crystal textiles with Janus structures for highly efficient personal thermal management. Composites Part B: Engineering, 2025, 297: 112344.

[2] Chai J, Wang G, Shao R, et al. Multifaceted Janus textile simultaneously achieving self-sustainable thermal management, perception, and protection. Nano-Micro Letters, 2026, 18(1): 205.

[3] Zhang Y, Qin W, Wang Y, et al. Stretchability-adjustable Janus composite fiber membrane for passive radiant cooling and heating. Energy and Buildings, 2024, 312: 114258.

[4] Zhou J, Zeng Q, Liu Y, et al. Bio-inspired dual-mode Janus film with optical adaptation for spatial thermal management and year-round energy saving. Nano Energy, 2025, 134: 110580.

[5] Dai B, Li X, Xu T, et al. Radiative cooling and solar heating janus films for personal thermal management. ACS applied materials & interfaces, 2022, 14(16): 18877-18883.

[6] Pian S, Wang Z, Lu C, et al. Scalable colored Janus fabric scheme for dynamic thermal management. Iscience, 2024, 27(10).

[7] Guo H, Li C, Yu J, et al. Tailored fabrics with biomimetic Janus spectral responsiveness for all-weather switchable thermoregulation. Advanced Functional Materials, 2024, 34(45): 2406638.

[8] Lan C, Meng J, Pan C, et al. Hierarchical porous dual-mode thermal management fabrics achieved by regulating solar and body radiations. Materials Horizons, 2024, 11(7): 1760-1768.

[9] Zhang J, Dong H, Yu Y, et al. An eco-friendly and multifunctional textile integrating radiative heating and cooling for large-temperature-variation personal thermal management. Advanced Functional Materials, 2025, 35(24): 2419777.

[10] Zhang R, Li Y, Yao B, et al. An electrospinning flexible textile integrating radiative cooling and solar heating for dynamic thermoregulation. Journal of Materials Chemistry A, 2025, 13(45): 38878-38886.
